# Information Graphs Incorporating Predictive Values of Disease Forecasts

**DOI:** 10.3390/e22030361

**Published:** 2020-03-20

**Authors:** Gareth Hughes, Jennifer Reed, Neil McRoberts

**Affiliations:** 1 SRUC, The King’s Buildings, Edinburgh EH9 3JG, UK; 2 Department of Plant Pathology, University of California, Davis, CA 95616, USA; reedje8@gmail.com (J.R.); nmcroberts@ucdavis.edu (N.M.)

**Keywords:** probability, forecast, likelihood ratio, positive predictive value, negative predictive value, diagnostic information, relative entropy

## Abstract

Diagrammatic formats are useful for summarizing the processes of evaluation and comparison of forecasts in plant pathology and other disciplines where decisions about interventions for the purpose of disease management are often based on a proxy risk variable. We describe a new diagrammatic format for disease forecasts with two categories of actual status and two categories of forecast. The format displays relative entropies, functions of the predictive values that characterize expected information provided by disease forecasts. The new format arises from a consideration of earlier formats with underlying information properties that were previously unexploited. The new diagrammatic format requires no additional data for calculation beyond those used for the calculation of a receiver operating characteristic (ROC) curve. While an ROC curve characterizes a forecast in terms of sensitivity and specificity, the new format described here characterizes a forecast in terms of relative entropies based on predictive values. Thus it is complementary to ROC methodology in its application to the evaluation and comparison of forecasts.

## 1. Introduction

Forecasting using two categories of actual status and two categories of forecast is common in many scientific and technical applications where evidence-based risk assessment is required as a basis for decision-making, including plant pathology and clinical medicine. The statistical evaluation of probabilistic disease forecasts often involves the calculation of metrics defined conditionally on actual disease status. For the purpose of disease management decision making, metrics defined conditionally on forecast outcomes (i.e., predictive values) are also of interest, although these are less frequently reported. Here we introduce a new diagrammatic format for disease forecasts with two categories of actual status and two categories of forecast. The format displays relative entropies, functions of predictive values that characterize expected information provided by disease forecasts. Our aims in introducing a new diagrammatic format are two-fold. First, we wish to highlight that performance metrics conditioned on forecast outcomes have a useful role in the overall evaluation of diagnostic tests and disease forecasters; second, bearing in mind the first aim, we wish to demonstrate that performance metrics based on information theoretic quantities can help distinguish characteristics of such tests and forecasters that may not be apparent from probability-scale metrics. The new diagrammatic format we introduce is intended to provide a generic approach that can applied in any suitable context.

Diagrammatic formats are useful for summarizing the processes of evaluation and comparison of disease forecasts in plant pathology and other disciplines where decisions about a subject must often be taken based on a proxy risk variable rather than knowledge of a subject’s actual status. The receiver operating characteristic (ROC) curve [1] is one such well-known format. In plant pathology, ROC curves are widely applied to characterize disease forecasters in terms of probabilities defined conditionally on actual disease status. Calculating the new diagrammatic format that we describe here has the same data requirements as the calculation of the ROC curve, but relates to relative entropy, an information theoretic metric that quantifies the expected amount of diagnostic information consequent on probability revision from prior to posterior arising from application of a disease forecaster. That is to say, it depicts (functions of) probabilities defined conditionally on the forecast. Even when the full underlying ROC curve data are not available, the new format can be constructed simply from ROC curve summary statistics.

The new diagrammatic format is linked analytically to other formats in ways that may not always be obvious simply from the resulting diagrams. We describe other formats and the links between them and the new format, using example data from a previously published study. In a general discussion, we consider the complementarity of metrics defined conditionally on the actual disease status and metrics defined conditionally on the outcome of the forecast. 

## 2. Methods 

We discuss information graphs for disease forecasters with two categories of actual status for subjects and two categories of forecast. In the present article, the terms ‘forecast’ and ‘prediction’ are synonymous. We place our discussion in the context of plant pathology, but the information graphs we describe likely have wider application. We are not concerned here with the detailed experimental and analytical methodology that underlies the development of disease forecasters. Readers seeking a description of such work are referred to Yuen et al. [2], Twengström et al. [3], and Yuen and Hughes [4], for example. Rather, we will describe some graphical methods for the comparison and evaluation of forecasters, and will outline some terminology and notation accordingly. 

We need forecasters for support in crop protection decision making because the stage of the growing season at which disease management decisions are taken is usually much earlier than an assessment of actual (or ‘gold standard’) disease status could be made. For the purpose of development of a forecaster, two disease assessments are made on each of a series of experimental crops during the growing season. The actual status of each crop is characterized by an assessment of yield, or of disease intensity, at the end of the growing season. Crops are classified as cases (‘*c*’) or non-cases (‘*nc*’), based on whether or not the gold standard end-of-season assessment indicates economically significant damage, respectively. Because the end-of-season assessment takes place too late to provide a basis for crop protection decision-making, an earlier assessment of disease risk is made, at a stage of the growing season when appropriate action can still be taken, if necessary. This earlier risk assessment may take the form of observation of a single variable that provides a risk score for the crop in question, or observation of a set of variables that are then combined to provide a risk score [5]. The risk score is a proxy variable, related to the actual status of the crop, that can be obtained at an appropriately early stage of the growing season for use in crop protection decision-making. Risk scores are usually calibrated so that higher scores are indicative of greater risk. 

Now, consider the introduction of a threshold on the risk score scale. Scores above the threshold are designated ‘+’, indicative of (predicted) need for a crop protection intervention. Scores at or below the threshold are designated ‘−’, indicative of (predicted) no need for a crop protection intervention. The considerations underlying the adoption of a specific threshold risk score for use in a particular crop protection setting are beyond the scope of this article. Madden [6] discusses this in connection with an example data set that we consider in more detail below. In all settings, an adopted threshold characterizes the operational classification rule that is used as a basis for predictions of the need or otherwise for a crop protection intervention. The variable that characterizes the risk score together with the adopted threshold risk score that characterizes the operational classification rule together characterize what we may refer to as a (binary) ‘test’ (‘forecaster’ and ‘predictor’ are synonymous). A prediction-realization table [7] encapsulates the cross-classified experimental data underlying such a test. The data provide estimates of probabilities as shown in Table 1. Then, from Table 1 via Bayes’ Rule, we can write ${\hat{p}}_{i\cap j}\;=\left[{\hat{p}}_{j\cap i}\right]\;=\;{\hat{p}}_{i|j}\cdot {\hat{p}}_{j}\;=\;{\hat{p}}_{j|i}\cdot {\hat{p}}_{i}$, with *i* = +, − (for the predictions) and *j* = *c*, *nc* (for the realizations). The ${\hat{p}}_{j}$ are taken as the Bayesian prior probabilities of case (*j* = *c*) or non-case (*j* = *nc*) status, such that ${\hat{p}}_{nc}=1-{\hat{p}}_{c}$. Note also that the ${\hat{p}}_{i}$ for intervention required (*i* = +) and intervention not required (*i* = −) can be written as ${\hat{p}}_{i}={\hat{p}}_{i|c}\cdot {\hat{p}}_{c}+{\hat{p}}_{i|nc}\cdot {\hat{p}}_{nc}$ via the Law of Total Probability.

The posterior probability of (gold standard) case status (*c*) given a + prediction on using a test is *p_c_*_|+_, referred to as the *positive predictive value*. Here, this refers to correct predictions of the need for a crop protection intervention; the complement *p_nc_*_|+_ = 1 − *p_c_*_|+_ refers to incorrect predictions of the need for an intervention. The posterior probability of (gold standard) non-case (*nc*) status given a – prediction on using a test is *p_nc_*_|−_, referred to as the *negative predictive value*. Here, this refers to correct predictions of no need for an intervention; the complement *p_c_*_|−_ = 1 − *p_nc_*_|−_ refers to incorrect predictions of no need for an intervention. If we think of *p_j_* (*j* = *c*, *nc*) as representing the Bayesian prior probabilities (i.e., before the test is used to make a prediction), the *p_j_*_|_*_i_* (*i* = +, −) then represent the corresponding posteriors (i.e., after obtaining the prediction). Predictive values are metrics defined conditionally on forecast outcomes.

The proportion of + predictions made for cases is referred to as the true positive proportion, or *sensitivity*, and provides an estimate of the conditional probability *p*_+|*c*_. The complementary false negative proportion is an estimate of *p*_−|*c*_. The proportion of + predictions made for non-cases is referred to as the false positive proportion, and provides an estimate of *p*_+|*nc*_. The complementary true negative proportion, or *specificity*, is an estimate of *p*_−|*nc*_. *Sensitivity* and *specificity* are metrics defined conditionally on actual disease status. The ROC curve, which has become a familiar device in crop protection decision support following the pioneering work of Jonathan Yuen and colleagues [2,3], is a graphical plot of *sensitivity* against 1−*specificity* for a set of possible binary tests, based on the disease assessments made during the growing season and derived by varying the threshold on the risk score scale. Since *sensitivity* and *specificity* values are linked, a disease forecaster based on a particular threshold represents values chosen to achieve an appropriate balance [8].

## 3. Results

### 3.1. Biggerstaff’s Analysis

We denote the likelihood ratio of a + prediction as $L_{+}$, estimated by:

(1) $${\hat{L}}_{+}=\frac{{\hat{p}}_{+}{|}_{\;c}}{{\hat{p}}_{+}{|}_{\;nc}}$$

(in words, the expression on the RHS is the true positive proportion divided by the false positive proportion or *sensitivity*/(1–*specificity*)). We denote the likelihood ratio of a − prediction as $L_{-}$, estimated by:

(2) $${\hat{L}}_{-}=\frac{{\hat{p}}_{-}{|}_{\;c}}{{\hat{p}}_{-}{|}_{\;nc}}$$

(in words, the expression on the RHS is the false negative proportion divided by the true negative proportion or (1–*sensitivity*)/*specificity*). Likelihood ratios are properties of a predictor (i.e., they are independent of prior probabilities) [9]. Values $L_{+}>1$ and $0<L_{-}<1$ are the minimum requirements for a useful binary test; within these ranges, larger positive values of $L_{+}$ and smaller positive values of $L_{-}$ are desirable. $L_{+}$ characterizes the extent to which a + prediction is more likely from *c* crops than from *nc* crops; $L_{-}$ characterizes the extent to which a − prediction is less likely from *c* crops than from *nc* crops.

Now, working in terms of odds (*o*) rather than probability (*p*) (with *o* = *p*/(1−*p*)), we can write versions of Bayes’ Rule, for example:

(3) $${\hat{o}}_{c|+}={\hat{o}}_{c}\cdot {\hat{L}}_{+}$$

and:

(4) $${\hat{o}}_{c|-}={\hat{o}}_{c}\cdot {\hat{L}}_{-}.$$

Thus, a + prediction increases the posterior odds of *c* status relative to the prior odds by a factor of ${\hat{L}}_{+}$ and a – prediction decreases the posterior odds of *c* status relative to the prior odds by a factor of ${\hat{L}}_{-}$. Biggerstaff [10] used Equations (3) and (4) to make pairwise comparisons of binary tests (with both tests applied at the same prior odds), premised on the availability only of the sensitivities and specificities corresponding to the two tests’ operational classification rules (for example, when considering tests for application based on their published ROC curve summary statistics, *sensitivity* and *specificity*).

At this point, we refer to a previously published phytopathological data set [11] in order to illustrate our analysis. Note, however, that the analysis we present is generic, and is not restricted to application in one particular pathosystem. Table 2 summarizes data for five different scenarios, based in essence on five different normalized prediction-realization tables, derived from the original data set and discussed previously in [6] in the context of decision making in epidemiology.

**Figure 1:**
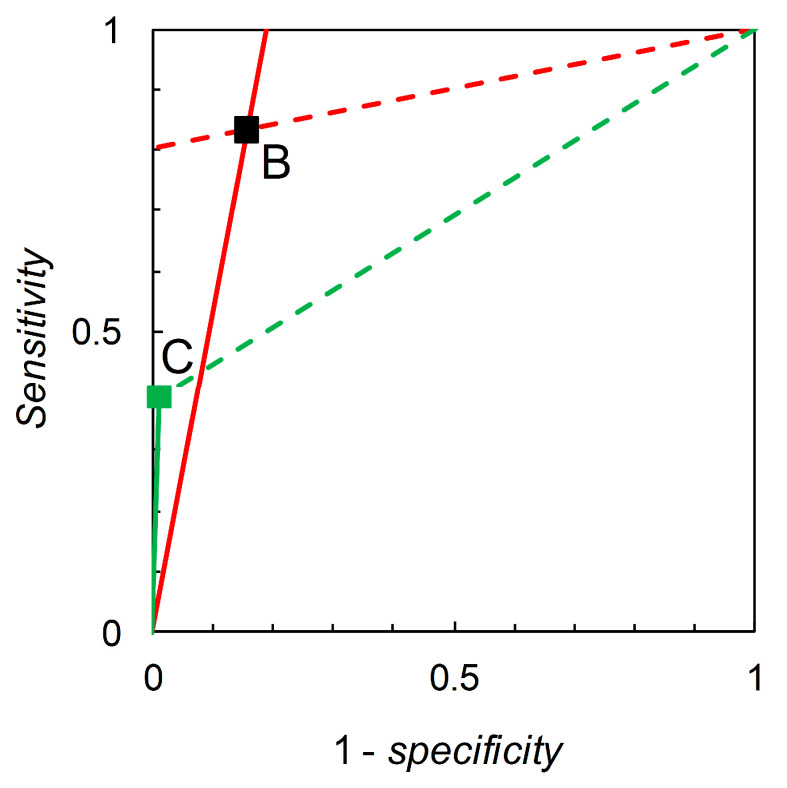
Biggerstaff’s likelihood ratios graph for Scenario B (reference) and Scenario C (comparison). The graph for Scenario B consists of a single point at 1–*specificity* = 0.156, *sensitivity* = 0.833 (see Table 2). The solid red line through (0, 0) and (0.156, 0.833) has slope = *sensitivity*/(1–*specificity*) = 5.333 = ${\hat{L}}_{+}$. The dashed red line through (0.156, 0.833) and (1, 1) has slope = (1–*sensitivity*)/*specificity* = 0.198 = ${\hat{L}}_{-}$. The graph for Scenario C consists of a single point at 1–*specificity* = 0.01, *sensitivity* = 0.39 (see Table 2). The solid green line through (0.01, 0.39) and (1, 1) has slope = *sensitivity*/(1–*specificity*) = 39.0 = ${\hat{L}}_{+}$. The dashed green line through (0.156, 0.833) and (1, 1) has slope = (1–*sensitivity*)/*specificity* = 0.616 = ${\hat{L}}_{-}$.

**Figure 2:**
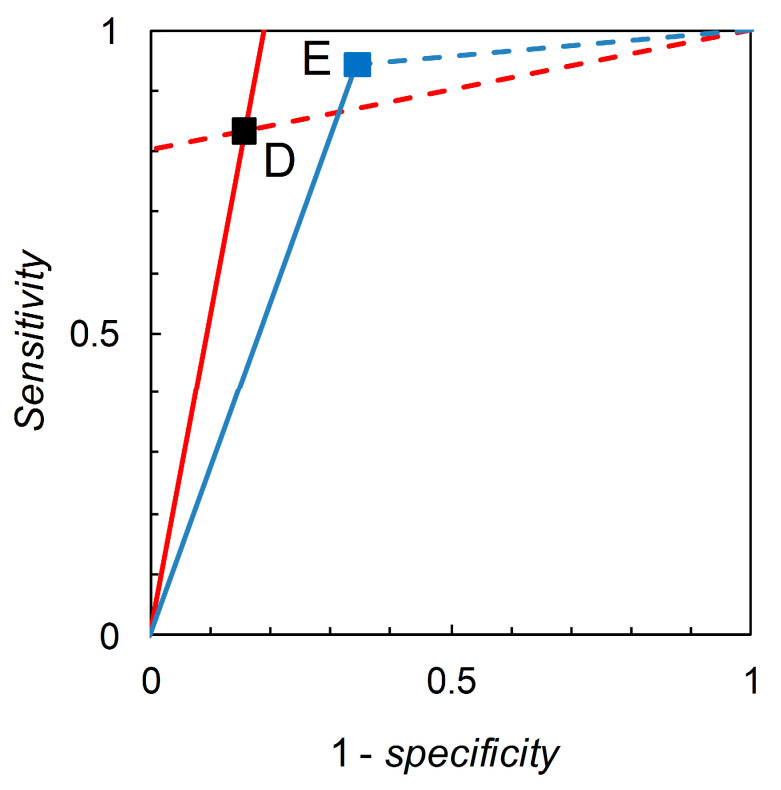
Biggerstaff’s likelihood ratios graph for Scenario D (reference) and Scenario E (comparison). The graph for Scenario D consists of a single point at 1–*specificity* = 0.156, *sensitivity* = 0.833 (see Table 2). The solid red line through (0, 0) and (0.156, 0.833) has slope = *sensitivity*/(1–*specificity*) = 5.333 = ${\hat{L}}_{+}$. The dashed red line through (0.156, 0.833) and (1, 1) has slope = (1–*sensitivity*)/*specificity* = 0.198 = ${\hat{L}}_{-}$. The graph for Scenario E consists of a single point at 1–*specificity* = 0.344, *sensitivity* = 0.944 (see Table 2). The solid blue line through (0, 0) and (0.344, 0.944) has slope = *sensitivity*/(1–*specificity*) = 2.744 = ${\hat{L}}_{+}$. The dashed blue line through (0.344, 0.944) and (1, 1) has slope = (1–*sensitivity*)/*specificity* = 0.085 = ${\hat{L}}_{-}$.

Recall that we are interested in probability (or odds) revision calculated on the basis of a forecast. For illustration, we first consider the pairwise comparison of the tests derived from Scenario B (reference) and Scenario C (comparison) made at ${\hat{p}}_{c}$ = 0.05 (Table 2). Madden [6] gives a detailed comparison based on knowledge of the full ROC curve derived from field experimentation. Biggerstaff’s analysis essentially represents an attempt to reverse engineer a similar comparison based only on knowledge of the tests’ published sensitivities and specificities. Scenario B yields *sensitivity* = 0.833 and *specificity* = 0.844, so we have ${\hat{L}}_{+}$ = 5.333 and ${\hat{L}}_{-}$ = 0.198. Scenario C yields *sensitivity* = 0.390 and *specificity* = 0.990, so we have ${\hat{L}}_{+}$ = 39.000 and ${\hat{L}}_{-}$ = 0.616. Thus, Scenario C’s test is superior in terms of ${\hat{L}}_{+}$ values but inferior in terms of ${\hat{L}}_{-}$ values (even though its *sensitivity* is lower and *specificity* higher than that of the reference test). As long as we restrict ourselves to pairwise comparisons of binary tests at the same prior probability we have a simple analysis that leads, via calculation of likelihood ratios, to an evaluation of tests made on the basis of Bayesian posteriors (directly in terms of posterior odds, but these are easily converted to posterior probabilities if so desired). The diagrammatic version of this comparison is shown in Figure 1. The likelihood ratios graph comprises two single-point ROC curves. A similar analysis for Scenario D (reference) and Scenario E (comparison) (Figure 2) shows that Scenario E’s test is inferior in terms of ${\hat{L}}_{+}$ values but superior in terms of ${\hat{L}}_{-}$ values (even though its *sensitivity* is higher and *specificity* lower than that of the reference test). 

Referring back to Table 2, the likelihood ratios, and corresponding graphs, for Scenarios A, B and D would be numerically identical. It is in this context that the information theoretic properties of likelihood ratios graphs (not pursued by Biggerstaff) are of interest. To elaborate further, we will require an estimate of the prior probability ${\hat{p}}_{c}$. This is beyond what Biggerstaff’s analysis allowed, but it is not so unlikely that such an estimate might be available. For example, a ${\hat{p}}_{c}$ value is provided for any test for which a numerical version of the prediction-realization table (see Table 1) is accessible. 

For information quantities, the specified unit depends on the choice of logarithmic base; bits for log base 2, nats for log base *e*, and hartleys (abbreviation: Hart) for log base 10 [12]. Our preference is to use base *e* logarithms, symbolized ln, where we need derivatives, following Thiel [7]. In this article, we will also make use of base 10 logarithms, symbolized log_10_, where this serves to make our presentation straightforwardly compatible with previously published work, specifically that of Johnson [13]. To convert from hartleys to nats, divide by log_10_(*e*); or to convert from nats to hartleys, divide by ln(10). When logarithms are symbolized just by log, as immediately following, this indicates use of a generic format such that specification of a particular logarithmic base is not required until the formula in question is used in calculation.

We start with disease prevalence as an estimate of the prior probability ${\hat{p}}_{c}$ of need for a crop protection intervention, and seek to update this by application of a predictor. The information required for certainty (i.e., when the posterior probability of need for an intervention is equal to one) is then $\log\left(1/{\hat{p}}_{c}\right)$ denominated in the appropriate information units. However, a predictor typically does not provide certainty, but instead updates ${\hat{p}}_{c}$ to ${\hat{p}}_{c|i}$< 1. The information still required for certainty is then $\log\left(1/{\hat{p}}_{c|i}\right)$ in the appropriate information units. We see from $\log\left(1/{\hat{p}}_{c}\right)-\log\left(1/{\hat{p}}_{c|\;i}\right)=\log\left({\hat{p}}_{c|\;i}/{\hat{p}}_{c}\right)$ that the term $\log\left({\hat{p}}_{c|\;i}/{\hat{p}}_{c}\right)$ represents the information content of prediction *i* in relation to actual status *c* in the appropriate information units. Provided the prediction is correct (i.e., in this case, *i* = +), the posterior probability is larger than the prior, and thus information content of the *positive predictive value* is > 0. In general, the information content of correct predictions is > 0. Predictions that result in a posterior unchanged from the prior have zero information content and incorrect predictions have information content < 0. 

Here, we consider the information content of a particular forecast, averaged over the possible actual states. These quantities are *expected* information contents, often referred to as relative entropies. For a binary test:

(5) $${\hat{I}}_{+}=\sum_{c,nc}{\hat{p}}_{j}{|}_{\;+}\cdot \log\left[\frac{{\hat{p}}_{j}{|}_{\;+}}{{\hat{p}}_{j}}\right]$$

for the forecast *i* = + and:

(6) $${\hat{I}}_{-}=\sum_{c,nc}{\hat{p}}_{j}{|}_{\;-}\cdot \log\left[\frac{{\hat{p}}_{j}{|}_{\;-}}{{\hat{p}}_{j}}\right]$$

for the forecast *i* = –. Relative entropies measure expected information consequent on probability revision from prior ${\hat{p}}_{j}$ to posterior ${\hat{p}}_{j|i}$ after obtaining a forecast. Relative entropies are ≥ 0, with equality only if the posterior probabilities are the same as the priors. Larger values of both ${\hat{I}}_{+}$ and ${\hat{I}}_{-}$ are preferable, as being indicative of forecasts that, on average, provide more diagnostic information.

**Figure 3:**
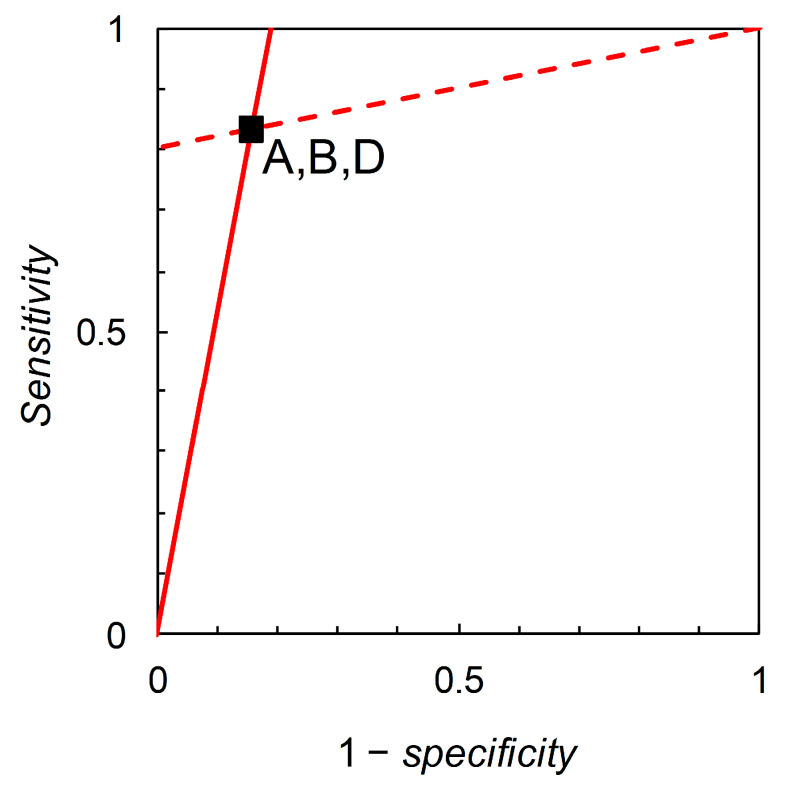
Biggerstaff’s likelihood ratios graphs for Scenarios A, B and D (Table 2). The slopes of the lines are the likelihood ratios ${\hat{L}}_{+}$ = 5.333 and ${\hat{L}}_{-}$ = 0.198, calculated from Table 2. Analysis shows that the lines themselves are also iso-information contours for the expected information contents of + and – forecasts. However, the calculated values of these expected information contents depend on the prior probability as well as on *sensitivity* and *specificity*. Making use of the available data on the prior probabilities allows us to calculate relative entropies in order to distinguish analytically between scenarios, but the likelihood ratios graph does not distinguish visually between scenarios with the same *sensitivity* and *specificity*.

We can write the relative entropies ${\hat{I}}_{+}$ and ${\hat{I}}_{-}$ in terms of *sensitivity*, *specificity* and (constant) prior probability. Working here in natural logarithms, and recalling that ${\hat{p}}_{-}{|}_{\;c}=1-{\hat{p}}_{+}{|}_{\;c}$, ${\hat{p}}_{-}{|}_{\;nc}=1-{\hat{p}}_{+}{|}_{\;nc}$, and ${\hat{p}}_{nc}=1-{\hat{p}}_{c}$ we have:

(7) $$\begin{array}{cc} {\hat{I}}_{+} & =\frac{{\hat{p}}_{+}{|}_{\;c}\cdot {\hat{p}}_{c}}{{\hat{p}}_{+}{|}_{\;c}\cdot {\hat{p}}_{c}+{\hat{p}}_{+}{|}_{\;nc}\cdot {\hat{p}}_{nc}}\cdot \ln\left[\frac{{\hat{p}}_{+}{|}_{\;c}}{{\hat{p}}_{+}{|}_{\;c}\cdot {\hat{p}}_{c}+{\hat{p}}_{+}{|}_{\;nc}\cdot {\hat{p}}_{nc}}\right]\\ & +\frac{{\hat{p}}_{+}{|}_{\;nc}\cdot {\hat{p}}_{nc}}{{\hat{p}}_{+}{|}_{\;c}\cdot {\hat{p}}_{c}+{\hat{p}}_{+}{|}_{\;nc}\cdot {\hat{p}}_{nc}}\cdot \ln\left[\frac{{\hat{p}}_{+}{|}_{\;nc}}{{\hat{p}}_{+}{|}_{\;c}\cdot {\hat{p}}_{c}+{\hat{p}}_{+}{|}_{\;nc}\cdot {\hat{p}}_{nc}}\right] \end{array}$$

in nats and:

(8) $$\begin{array}{cc} {\hat{I}}_{-} & =\frac{{\hat{p}}_{-}{|}_{\;c}\cdot {\hat{p}}_{c}}{{\hat{p}}_{-}{|}_{\;c}\cdot {\hat{p}}_{c}+{\hat{p}}_{-}{|}_{\;nc}\cdot {\hat{p}}_{nc}}\cdot \ln\left[\frac{{\hat{p}}_{-}{|}_{\;c}}{{\hat{p}}_{-}{|}_{\;c}\cdot {\hat{p}}_{c}+{\hat{p}}_{-}{|}_{\;nc}\cdot {\hat{p}}_{nc}}\right]\\ & +\frac{{\hat{p}}_{-}{|}_{\;nc}\cdot {\hat{p}}_{nc}}{{\hat{p}}_{-}{|}_{\;c}\cdot {\hat{p}}_{c}+{\hat{p}}_{-}{|}_{\;nc}\cdot {\hat{p}}_{nc}}\cdot \ln\left[\frac{{\hat{p}}_{-}{|}_{\;nc}}{{\hat{p}}_{-}{|}_{\;c}\cdot {\hat{p}}_{c+}{\hat{p}}_{-}{|}_{\;nc}\cdot {\hat{p}}_{nc}}\right] \end{array}$$

again in nats. Now we can use these formulas to plot sets of iso-information contours for constant relative entropies ${\hat{I}}_{+}$ and ${\hat{I}}_{-}$ on the graph with axes *sensitivity* and 1 – *specificity*, for given prior probabilities. From Equation (7) we obtain:

(9) $$\frac{d\left({\hat{p}}_{+}{|}_{\;c}\right)}{d\left({\hat{p}}_{+}{|}_{\;nc}\right)}=\frac{{\hat{p}}_{+}{|}_{\;c}}{{\hat{p}}_{+}{|}_{\;nc}}$$

the solution of which is the straight line ${\hat{p}}_{+}{|}_{\;c}=a\cdot {\hat{p}}_{+}{|}_{\;nc}$, which yields $a={\hat{L}}_{+}$. From Equation (8) we obtain:

(10) $$\frac{d\left({\hat{p}}_{+}{|}_{\;c}\right)}{d\left({\hat{p}}_{+}{|}_{\;nc}\right)}=\frac{1-{\hat{p}}_{+}{|}_{\;c}}{1-{\hat{p}}_{+}{|}_{\;nc}}$$

the solution of which is the straight line ${\hat{p}}_{+}{|}_{\;c}=\left(1-b\right)+b\cdot {\hat{p}}_{+}{|}_{\;nc}$, which yields $b={\hat{L}}_{-}$. Thus, we find that iso-information contours for ${\hat{I}}_{+}$ and ${\hat{I}}_{-}$ are straight lines on the graph with axes *sensitivity* and 1 – *specificity*, i.e., Biggerstaff’s likelihood ratios graph (see Figure 3).

Now consider Scenarios A, B and D; from the data in Table 2, we calculate likelihood ratios ${\hat{L}}_{+}$ = 5.333 and ${\hat{L}}_{-}$ = 0.198 for all three scenarios (these are the slopes of the lines shown in Figure 3). However, the three scenarios differ in their prior probabilities: ${\hat{p}}_{c}$ = 0.36, 0.05, 0.85 for A, B, and D respectively. This situation may arise in practice when a test is developed and used in one geographical location, and then subsequently evaluated with a view to application in other locations where the disease prevalence is different. The difference in test performance is reflected by the relative entropy calculations. For Scenario A, we calculate relative entropies ${\hat{I}}_{+}$ = 0.315 and ${\hat{I}}_{-}$ = 0.179 (both in nats, these characterize the lines shown in Figure 3 interpreted as iso-information contours for the expected information contents of + and – forecasts respectively). For Scenario B, we calculate ${\hat{I}}_{+}$ = 0.171 and ${\hat{I}}_{-}$ = 0.024 nats. For Scenario D, ${\hat{I}}_{+}$ = 0.076 and ${\hat{I}}_{-}$ = 0.289 nats. Thus we may view Biggerstaff’s likelihood ratios graph from an information theoretic perspective. While likelihood ratios are independent of prior probability, relative entropies are functions of prior probability. There is further discussion of relative entropies, including calculations for Scenarios C and E, in Section 3.3.

### 3.2. Johnson’s Analysis

Johnson [13] suggested transformation of the likelihood ratios graph (e.g., Figure 1, Figure 2 and Figure 3), such that the axes of the graph are denominated in log likelihood ratios. At the outset, note that Johnson works in base 10 logarithms and that this choice is duplicated here, for the sake of compatibility. Thus, although Johnson’s analysis is not explicitly information theoretic, where we use it as a basis for characterizing information theoretic quantities, these quantities will have units of hartleys. Note also that Johnson calculates $\left|\log_{10}{\hat{L}}_{+}\right|$ and $\left|\log_{10}{\hat{L}}_{-}\right|$ but here we take account of the signs of the log likelihood ratios. Fosgate’s [14] correction of Johnson’s terminology is noted, although this does not affect our analysis at all.

From Equation (3), we write:

(11) $$\log_{10}{\hat{o}}_{c}{|}_{\;+}=\log_{10}{\hat{o}}_{c+}\log_{10}{\hat{L}}_{+}$$

and from Equation (4):

(12) $$\log_{10}{\hat{o}}_{c}{|}_{\;-}=\log_{10}{\hat{o}}_{c+}\log_{10}{\hat{L}}_{-}$$

with $\log_{10}{\hat{L}}_{+}$> 0 (larger positive values are better) and $\log_{10}{\hat{L}}_{-}$< 0 (larger negative values are better) for any useful test. As previously, the objective is to make pairwise comparisons of binary tests (with both tests applied at the same prior odds), premised on the availability only of the sensitivities and specificities corresponding to the two tests’ operational classification rules. 

**Figure 4:**
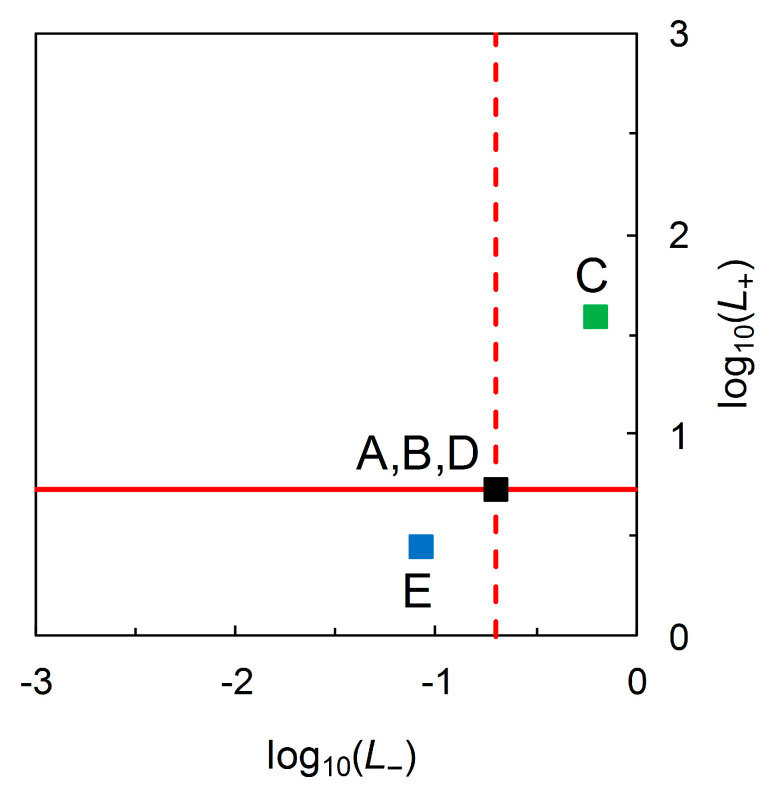
A version of Johnson’s log_10_ likelihood ratios diagram for data from Table 2. Here $\log_{10}{\hat{L}}_{+}$ = 0.727 and $\log_{10}{\hat{L}}_{-}$= −0.704 for Scenarios A, B and D (■). For Scenario C (■), $\log_{10}{\hat{L}}_{+}$ = 1.591 and $\log_{10}{\hat{L}}_{-}$= −0.208. For Scenario E (■), $\log_{10}{\hat{L}}_{+}$ = 0.438 and $\log_{10}{\hat{L}}_{-}$ = −1.071. Valid comparisons (i.e., for scenarios with equal prior probabilities) are Scenario B (reference) with Scenario C (comparison) and Scenario D (reference) with Scenario E (comparison).

With Scenario B as the reference test and Scenario C as the comparison test, we find Scenario C’s test is superior in terms of $\log10{\hat{L}}_{+}$ values but inferior in terms of $\log10{\hat{L}}_{-}$ values (Figure 4). With Scenario D as the reference test and Scenario E as the comparison test, we find Scenario E’s test is inferior in terms of $\log10{\hat{L}}_{+}$ values, but superior in terms of $\log10{\hat{L}}_{-}$ (Figure 4). Moreover, we find that the transformed likelihood ratios graph still does not distinguish visually between Scenarios A, B and D (Figure 4). Thus, the initial findings from the analysis of the scenarios in Table 2 are the same as previously. 

**Figure 5:**
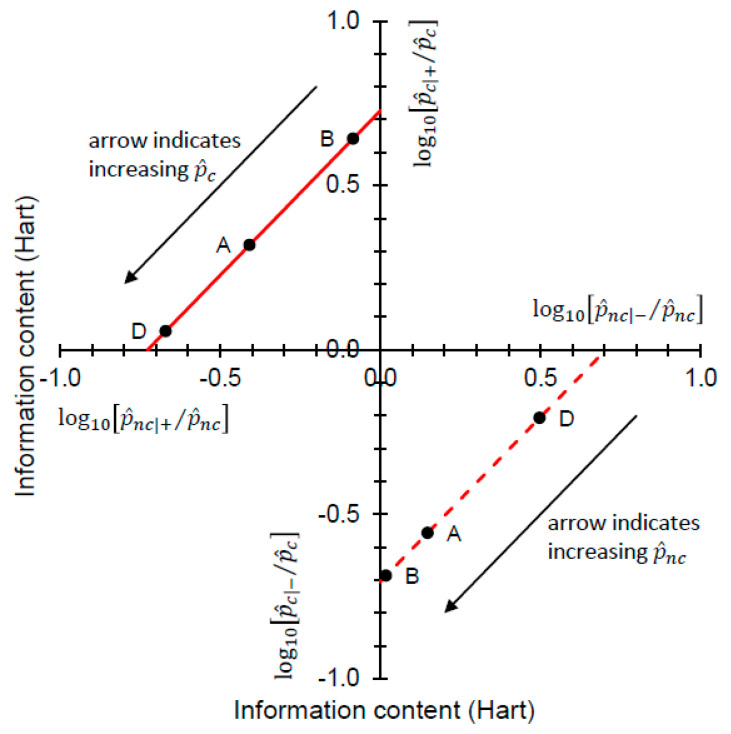
The “north-west” region of the figure is characterized by Equation (13), so relates to + predictions (which are correct for *c* subjects and incorrect for *nc* subjects). ${\mathrm{Log}}_{10}L_{+}$ contours are always straight lines with slope = 1. The solid red line indicates the contour for $\log_{10}{\hat{L}}_{+}$ = 0.727 Hart, corresponding to Scenarios A, B, and D (Table 2). A correct + prediction has a large information content when ${\hat{p}}_{c}$ is small (B), and a small information content is when ${\hat{p}}_{c}$ is large (D) (the arrow indicates the direction of increasing ${\hat{p}}_{c}$ along the contour). As the information content $\log_{10}\left[{\hat{p}}_{c}{|}_{+}/{\hat{p}}_{c}\right]$ (on the vertical axis) becomes decreasingly positive, the information content $\log_{10}\left[{\hat{p}}_{nc}{|}_{+}/{\hat{p}}_{nc}\right]$ (on the horizontal axis) becomes increasingly negative. The “south-east” region of the figure is characterized by Equation (14), so relates to − predictions (which are correct for *nc* subjects and incorrect for *c* subjects). ${\mathrm{Log}}_{10}L_{-}$ contours are always straight lines with slope = 1. The dashed red line indicates the contour for $\log_{10}{\hat{L}}_{-}$ = −0.704 Hart, corresponding to Scenarios A, B, and D (Table 2). A correct − prediction has a large information content when ${\hat{p}}_{nc}$ is small (D), and a small information content is when ${\hat{p}}_{nc}$ is large (B) (the arrow indicates the direction of increasing ${\hat{p}}_{nc}$ along the contour, ${\hat{p}}_{nc}=1-{\hat{p}}_{c}$). As the information content $\log_{10}\left[{\hat{p}}_{nc}{|}_{-}/{\hat{p}}_{nc}\right]$ (on the horizontal axis) becomes decreasingly positive, the information content $\log_{10}\left[{\hat{p}}_{c}{|}_{-}/{\hat{p}}_{c}\right]$ (on the vertical axis) becomes increasingly negative.

Now, as with Biggerstaff’s [10] original analysis, we seek to view Johnson’s analysis from an information theoretic perspective. As before, we will require an estimate of the prior probability ${\hat{p}}_{c}$. After some rearrangement, we obtain from Equation (11):

(13) $$\log_{10}\left[\frac{{\hat{p}}_{c}{|}_{+}}{{\hat{p}}_{c}}\right]-\log_{10}\left[\frac{{\hat{p}}_{nc}{|}_{+}}{{\hat{p}}_{nc}}\right]=\log_{10}{\hat{L}}_{+}\mathrm{Hart}$$

where $\log_{10}\left[{\hat{p}}_{c}{|}_{+}/{\hat{p}}_{c}\right]$ (> 0) and $\log_{10}\left[{\hat{p}}_{nc}{|}_{+}/{\hat{p}}_{nc}\right]$ (< 0) on the LHS are information contents (as outlined in Section 3.1) with units of hartleys. From Equation (12):

(14) $$\log_{10}\left[\frac{{\hat{p}}_{c}{|}_{-}}{{\hat{p}}_{c}}\right]-\log_{10}\left[\frac{{\hat{p}}_{nc}{|}_{-}}{{\hat{p}}_{nc}}\right]=\log_{10}{\hat{L}}_{-}\mathrm{Hart}$$

where $\log_{10}\left[{\hat{p}}_{c}{|}_{-}/{\hat{p}}_{c}\right]$ (< 0) and $\log_{10}\left[{\hat{p}}_{nc}{|}_{-}/{\hat{p}}_{nc}\right]$ (> 0) on the LHS are information contents, again with units of hartleys. Thus, we recognize that log_10_ likelihood ratios also have units of hartleys. Figure 5 shows the information theoretic characteristics of Johnson’s analysis when data on priors are incorporated, by drawing log_10_-likelihood contours on a graphical plot that has information contents on the axes. 

In Figure 5, both the $\log_{10}{\hat{L}}_{+}$ and $\log_{10}{\hat{L}}_{-}$ contours always have slope = 1. As the decompositions characterized in Equations (13) and (14) show, any (constant) log_10_ likelihood ratio is the sum of two information contents. Looking at the “north-west” corner of Figure 5 and taking Scenarios A, B, and D from Table 2 as examples, we have $\log_{10}\left[{\hat{p}}_{c}{|}_{+}/{\hat{p}}_{c}\right]$ = 0.642, 0.319, 0.056 Hart and $\log_{10}\left[{\hat{p}}_{nc}{|}_{+}/{\hat{p}}_{nc}\right]$ = −0.085, −0.408, −0.671 Hart for ${\hat{p}}_{c}$ = 0.05 (B), 0.36 (A), 0.85 (D), respectively. In each case, Equation (13) yields $\log_{10}{\hat{L}}_{+}$ = 0.727 Hart. Looking at the “south-east” corner of Figure 5, again taking Scenarios A, B, and D from Table 2 as examples, we have $\log_{10}\left[{\hat{p}}_{nc}{|}_{-}/{\hat{p}}_{nc}\right]$ = 0.498, 0.148, 0.018 Hart and $\log_{10}\left[{\hat{p}}_{c}{|}_{-}/{\hat{p}}_{c}\right]$ = −0.207, −0.556, −0.687 Hart for ${\hat{p}}_{nc}$ = 0.15 (D), 0.64 (A), 0.95 (B), respectively. In each case, Equation (14) yields $\log_{10}{\hat{L}}_{-}$ = −0.704 Hart. Thus we have an information theoretic perspective on Johnson’s analysis when data on priors are available, and this time one that separates Scenarios A, B and D visually (Figure 5).

### 3.3. A New Diagrammatic Format

Biggerstaff’s [10] diagrammatic format for binary predictors allows an information theoretic interpretation once the data on prior probabilities have been incorporated. This distinguishes predictors with the same likelihood ratios analytically, but not visually. Johnson’s [13] transformed version of Biggerstaff’s diagrammatic format also allows an information theoretic interpretation once data on prior probabilities are incorporated. This approach distinguishes predictors with the same likelihood ratios both analytically and visually, but does not contribute to the comparison and evaluation of predictive values of disease forecasters. 

**Figure 6:**
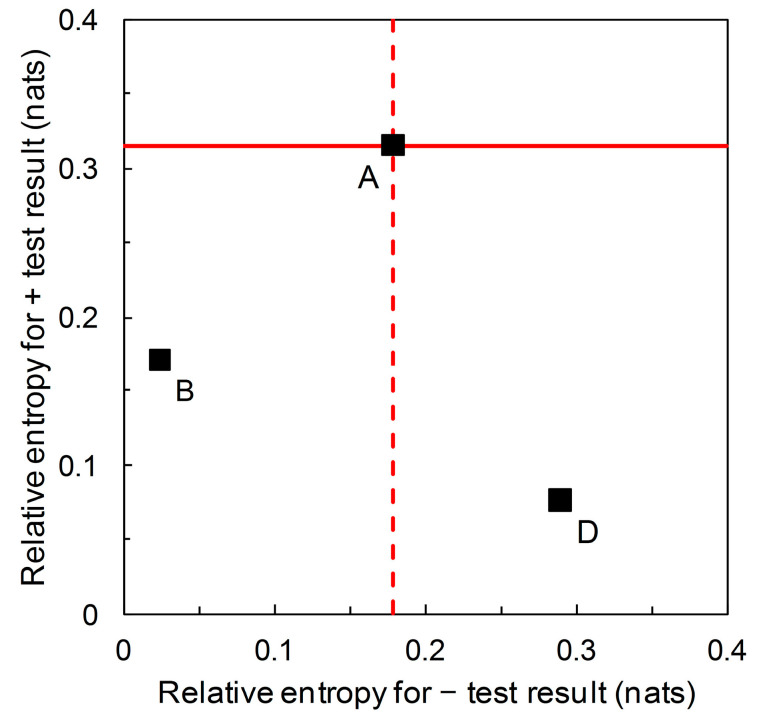
Scenario A: from the data in Table 2, we calculate relative entropies ${\hat{I}}_{+}$ = 0.315, ${\hat{I}}_{-}$ = 0.179 (both in nats) (${\hat{p}}_{c}$ = 0.36) (Equations (3) and (4)). Similarly, for Scenario B we calculate ${\hat{I}}_{+}$ = 0.171, ${\hat{I}}_{-}$ = 0.024 nats (${\hat{p}}_{c}$ = 0.05) and for Scenario D, ${\hat{I}}_{+}$ = 0.076, ${\hat{I}}_{-}$ = 0.289 nats (${\hat{p}}_{c}$ = 0.85).

We now return to the information theoretic interpretation of Biggerstaff’s likelihood ratios graph (and revert to working in natural logarithms for continuity with previous analysis based on Figure 3). In Figure 3, the likelihood ratios are the slopes of the lines on the graphical plot. The lines themselves are relative entropy contours, the value of which depends on prior probability. We can now visually separate scenarios that have the same likelihood ratios but different relative entropies (e.g., A, B, D in Table 2) by calculating the graph with relative entropies ${\hat{I}}_{+}$ and ${\hat{I}}_{-}$ on the axes of the plot (Figure 6). If we consider the predictor based on Scenario A as the reference, then the predictor based on Scenario B falls in the region of Figure 6 indicating comparatively less information is provided by both + and – predictions, while the predictor based on Scenario D falls in the region indicating comparatively less diagnostic information is provided by + predictions but comparatively more by − predictions. 

**Figure 7:**
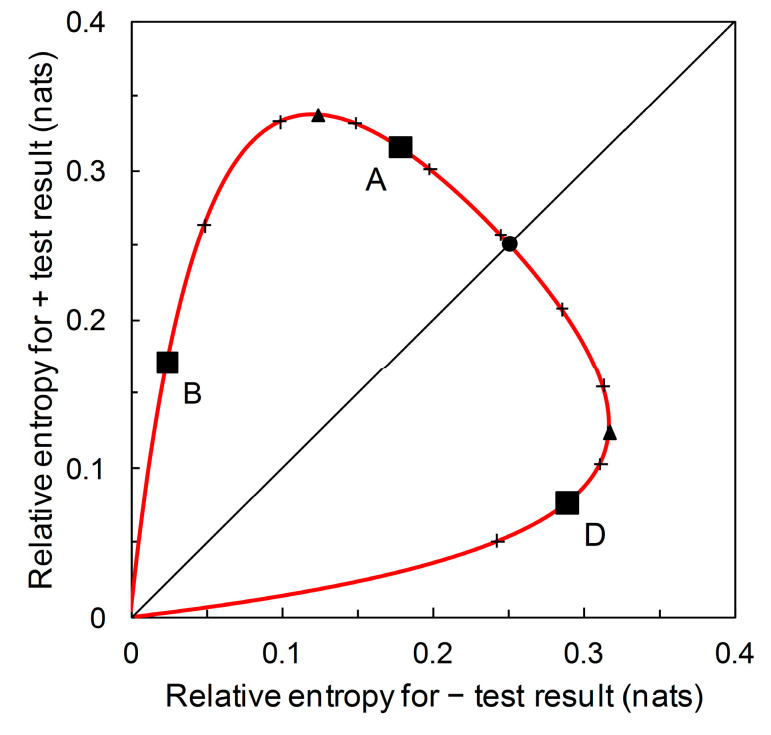
The prior probability ${\hat{p}}_{c}$ contour for Scenarios A, B, and D (solid red line). The contour is calibrated at 0.1 intervals of ${\hat{p}}_{c}$, clockwise from the origin, 0.1 to 0.9 (+ symbol on curve). Scenarios B (${\hat{p}}_{c}$ = 0.05), A (${\hat{p}}_{c}$ = 0.36), and D (${\hat{p}}_{c}$ = 0.85) as characterized in Table 2 are indicated (■). Also indicated on the prior probability contour: maximum ${\hat{I}}_{+}$ = 0.337 nats (▲) (${\hat{p}}_{c}$ = 0.245), maximum ${\hat{I}}_{-}$ = 0.317 nats (▲) (${\hat{p}}_{c}$ = 0.749), ${\hat{I}}_{+}$ = ${\hat{I}}_{-}$= 0.251 nats (●) (${\hat{p}}_{c}$ = 0.513).

There is an alternative view of the diagrammatic format presented in Figure 6. Scenarios A, B and D all have the same likelihood ratios, ${\hat{L}}_{+}$= 5.333 and ${\hat{L}}_{-}$ = 0.198 (see Figure 3). What differs between scenarios is the prior probability ${\hat{p}}_{c}$. We can remove the gridlines indicating the relative entropies for Scenario A and plot the underlying prior probability contour (Figure 7). In Figure 7, starting at the origin and moving clockwise, prior probability increases as we move along the contour. The contour has maximum points with respect to both the horizontal axis and the vertical axis. The maximum value of the contour with respect to the horizontal axis is:

(15) $${\hat{p}}_{c}=\frac{{\hat{p}}_{+}{|}_{\;nc}\cdot \left[{\hat{p}}_{+}{|}_{\;c}\cdot \left(\ln\left[\frac{{\hat{p}}_{+}{|}_{\;c}}{{\hat{p}}_{+}{|}_{\;nc}}\right]-1\right)+{\hat{p}}_{+}{|}_{\;nc}\right]}{{\left[{\hat{p}}_{+}{|}_{\;c}-{\hat{p}}_{+}{|}_{\;nc}\right]}^{2}}$$

and the maximum value of the contour with respect to the vertical axis is:

(16) $${\hat{p}}_{c}=\frac{{\hat{p}}_{-}{|}_{\;nc}\cdot \left[{\hat{p}}_{-}{|}_{\;c}\cdot \left(\ln\left[\frac{{\hat{p}}_{-}{|}_{\;c}}{{\hat{p}}_{-}{|}_{\;nc}}\right]-1\right)+{\hat{p}}_{-}{|}_{\;nc}\right]}{{\left[{\hat{p}}_{+}{|}_{\;c}-{\hat{p}}_{+}{|}_{\;nc}\right]}^{2}}.$$

The corresponding values of ${\hat{I}}_{+}$ and ${\hat{I}}_{-}$, respectively, can then be calculated by substitution into Equations (7) and (8). The two maxima (together with the origin) divide the prior probability contour into three monotone segments (see Figure 7). As ${\hat{p}}_{c}$ increases, we observe a segment where ${\hat{I}}_{+}$ and ${\hat{I}}_{-}$ are both increasing (this includes Scenario B), then one where ${\hat{I}}_{+}$ is decreasing and ${\hat{I}}_{-}$ is increasing, this includes Scenario A), and then one where ${\hat{I}}_{+}$ and ${\hat{I}}_{-}$ are both decreasing (this includes Scenario D).

From Figure 7, we see that for the predictor based on Scenarios A, B and D, a + prediction provides most diagnostic information around prior probability 0.2 < ${\hat{p}}_{c}$ < 0.3. A – prediction provides most diagnostic information around prior probability 0.7 < ${\hat{p}}_{c}$ < 0.8. Recall that this contour describes performance (in terms of diagnostic information provided) for predictors with *sensitivity* = 0.833 and *specificity* = 0.844 (Table 2) (alternatively expressed as likelihood ratios ${\hat{L}}_{+}$ = 5.333 and ${\hat{L}}_{-}$ = 0.198). No additional data beyond *sensitivity* and *specificity* are required in order to produce this graphical plot; that is to say, by considering the whole range of prior probability we remove the requirement for any particular values. The point where the contour intersects the main diagonal of the plot is where ${\hat{I}}_{+}$ = ${\hat{I}}_{-}$. In this case, we find that ${\hat{I}}_{+}$ = ${\hat{I}}_{-}$ at prior probability ≈ 0.5 (Figure 7). At lower prior probabilities, + predictions provide more diagnostic information than – predictions, while at higher prior probabilities, the converse is the case. This contour’s balance of relative entropies at prior probability ≈ 0.5 is noteworthy because it is not necessarily the case that there is always scope for such balance. 

**Figure 8:**
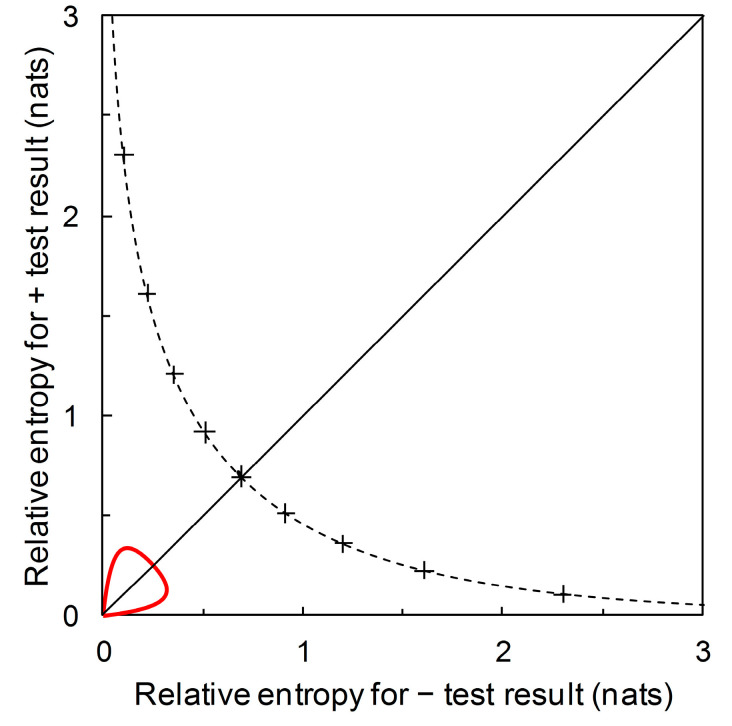
The dashed curve is the prior probability ${\hat{p}}_{c}$ contour showing the upper limit for performance of any binary predictor. The contour is calibrated at 0.1 intervals of ${\hat{p}}_{c}$ from upper left to lower right, 0.1 to 0.9 (+ symbol on curve). The maximum relative entropy for a + test result increases indefinitely as ${\hat{p}}_{c}$ approaches 0 while the maximum relative entropy for a – test result increases indefinitely as ${\hat{p}}_{c}$ approaches 1. The prior probability contour for Scenarios A, B, and D from Figure 7 (solid red line) is also shown, for reference (note the rescaled axes).

Recall from Section 3.1 that we start with disease prevalence as an estimate of the prior probability ${\hat{p}}_{c}$ of need for a crop protection intervention. The information required (from a predictor) for certainty is then $\log\left(1/{\hat{p}}_{c}\right)$ denominated in the appropriate information units. This is the amount of information that would result in a posterior probability of need for an intervention equal to one. Similarly, $\log\left(1/{\hat{p}}_{nc}\right)$, denominated in the appropriate information units, is the amount of information that would result in a posterior probability of no need for an intervention equal to one. We can plot the contour for these information contents on the diagrammatic format of Figure 7. This contour, illustrated in Figure 8, indicates the upper limit for the performance of any binary predictor. No phytopathological data are required to calculate this contour.

**Figure 9:**
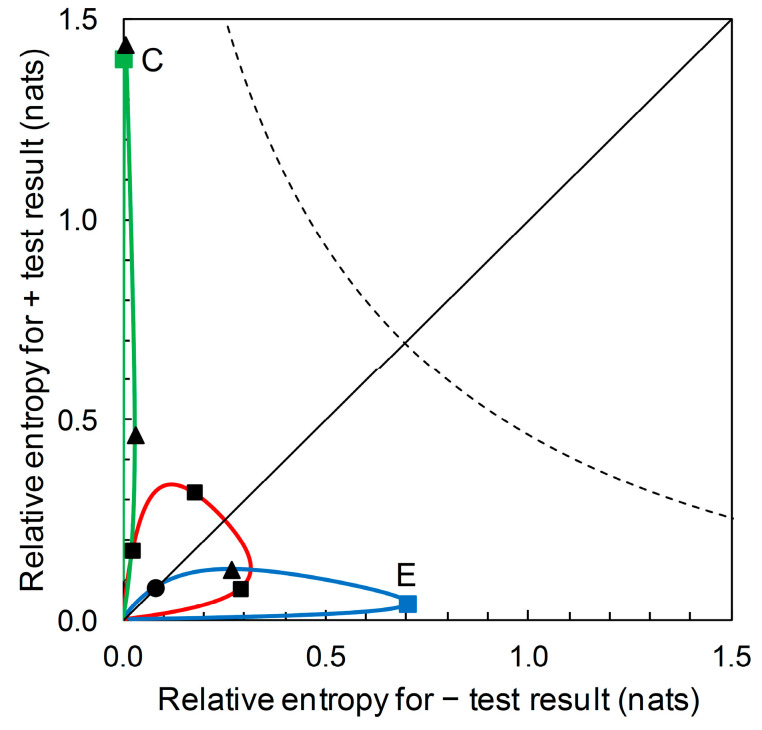
The prior probability contours for Scenarios C (solid green line) and E (solid blue line). Starting at the origin, the green prior probability contour passes through points (clockwise from origin): Scenario C, ${\hat{I}}_{+}$ = 1.399, ${\hat{I}}_{-}$ = 0.004 (prior = 0.05) (■); maximum ${\hat{I}}_{+}$ = 1.436 (prior = 0.073) (▲); maximum ${\hat{I}}_{-}$ = 0.029 (prior = 0.580) (▲). This contour does not coincide with the main diagonal of the plot other than at the origin. Starting at the origin, the blue prior probability contour passes through points (clockwise from origin): ${\hat{I}}_{+}$ = ${\hat{I}}_{-}$ = 0.080 (●) (prior = 0.109); maximum ${\hat{I}}_{+}$ = 0.126 (prior = 0.337) (▲); Scenario E, ${\hat{I}}_{+}$ = 0.039, ${\hat{I}}_{-}$ = 0.700 (prior = 0.850) (■); maximum ${\hat{I}}_{-}$ = 0.701 (prior = 0.842) (point obscured from view). The prior probability contour for Scenarios A, B, and D (solid red line) is included here for reference; clockwise from origin, points marked ■ indicate Scenarios B, A and D (see Figure 7 for details). The dashed curve shows the contour indicating the upper limit for performance of a binary predictor (see Figure 8 for details). Note the changes in the scales on the axes compared with Figure 7 and Figure 8.

The diagrammatic format of Figure 7 (for Scenarios A, B and D) can accommodate prior probability contours for other Scenarios (i.e., for predictors based on different *sensitivity* and *specificity* values). For example, Figure 9 shows, in addition, the prior probability contours for the predictors based on Scenario C (with *sensitivity* = 0.39 and *specificity* = 0.99) and on Scenario E (with *sensitivity* = 0.944 and *specificity* = 0.656). We observe that a predictor based on Scenario C’s *sensitivity* and *specificity* values potentially provides a large amount of diagnostic information from a + prediction, but over a very narrow range of prior probabilities. Scenario C itself represents one such predictor. The amount of diagnostic information from − predictions is very low over the whole range of prior probabilities. A predictor based on Scenario E’s *sensitivity* and *specificity* values potentially provides a large amount of diagnostic information from − predictions over a narrow range of prior probabilities. Scenario E itself represents one such predictor. The amount of diagnostic information from + predictions remains low over the whole range of prior probabilities.

## 4. Discussion

Diagrammatic formats have the potential to aid interpretation in the evaluation and comparison of disease forecasts. Biggerstaff’s [10] likelihood ratios graph is a particularly interesting example. This graph uses the format of the ROC curve, as widely applied in exhibiting and explaining *sensitivity* and *specificity* for binary tests. However, while *sensitivity* and *specificity* are defined conditionally on actual disease status, the likelihood ratios graph is used to compare tests on the basis of predictive values, defined conditionally on the forecast (when tests are applied at the same prior probability). As Biggerstaff notes, one is less interested in *sensitivity* and *specificity* when it comes to the application of a test, because the conditionality is in the wrong order. The predictive values, or some functions of them, are also important, and ideally one would be able use these when assessing test performance in application (Figure 1 and Figure 2). 

Altman and Royston [15] discussed this idea in some detail and proposed PSEP as a metric for use in the assessment of predictor performance (in the binary case, PSEP = *positive predictive value* + *negative predictive value* – 1). Hughes and Burnett [16] later used an information theoretic analysis (including a diagrammatic representation) to show how PSEP was related to both the *Brier score* [17] and the information theoretic *divergence score* [18] methods of assessing predictor performance. In the current article, further analysis shows that Biggerstaff’s likelihood ratios graph has underlying information theoretic properties that specifically relate to predictive values. The lines on the likelihood ratios graph are relative entropy contours, quantifying the expected information consequent on revising the prior probability of disease to the posterior probability after obtaining a forecast. However, the likelihood ratios graph does not visually distinguish relative entropy contours when predictors that have the same ROC curve summary statistics (sensitivities and specificities, or equivalently, likelihood ratios for both + and − predictions) are compared at different prior probabilities (Figure 3). A modified diagrammatic format that does so would therefore be of interest. 

Johnson [13] provides a modified format, with log likelihood ratios on the axes of the graph (Figure 4), and suggests various possible advantages of this format. Our further analysis again shows that this modified format has underlying information theoretic properties. These properties relate to the statistical decomposition of log likelihood ratios (Figure 5; see also [5] for further discussion) but do not appear to be straightforwardly helpful as an aid to interpretation in the evaluation and comparison of disease forecasters based on predictive values. 

Benish [19] applied information graphs for relative entropy to evaluate and compare clinical diagnostic tests. Here we derive relative entropies from Biggerstaff’s likelihood ratios graph and present the results in a new diagrammatic format, with relative entropies for + and − predictions on the axes of the graph. Compared with the likelihood ratios graph, this visually distinguishes between predictors that have the same ROC curve summary statistics when compared at different (known) prior probabilities (Figure 6). So, referring to the scenarios listed in Table 2 with likelihood ratios ${\hat{L}}_{+}$ = 5.333 and ${\hat{L}}_{-}$ = 0.198 (i.e., A, B, and D) we see that Scenario A has the highest relative entropy for a + prediction, then B, then D. Scenario D has the highest relative entropy for a − prediction, then A, then B. Recall that relative entropies are functions of the predictive values. 

Suppose now that our aim is not to compare predictor performance in particular scenarios, but to evaluate performance over the range of possible scenarios. We can use our new format not just to plot relative entropies for a comparison of predictor performance for various known prior probability (disease prevalence) scenarios (Figure 6), but to also draw the contour showing how relative entropies change as prior probability of disease varies over the range from zero to one (Figure 7). This diagrammatic format requires no particular prior probabilities for calculation, only the ROC curve summary statistics. In the same way that the ROC curve relates to all predictors (by *sensitivity* and *specificity*) until a particular operational threshold is set, Figure 7 relates to all predictors (by relative entropies based on predictive values) until a particular prior probability value is specified. Maximum relative entropy points on the contour are calculable analytically in this format. Moreover, we can include the contours for predictors with different summary statistics. Figure 9 shows the contour that includes the predictor based on Scenario C and the contour that includes the predictor based on Scenario E, in addition to the contour that includes predictors based on Scenarios A, B and D from Figure 7. In this diagrammatic format, we can easily see the difference between contours that include predictors with high performance (in terms of relative entropies) in a narrow range of applicability (in terms of prior probabilities) when compared with a contour that balances predictor performance with a wider range of applicability. Unless we wish to evaluate and/or compare particular scenarios—in which case, not unreasonably, estimates of the corresponding prior probability (disease prevalence) values are required—producing the contour plot (Figure 7 and Figure 9) has no data requirements beyond those for producing the ROC curve. 

Figure 8 and Figure 9 include the contour showing the upper limit for performance of a binary predictor. This upper limit serves as a qualitative visual calibration of predictor performance, rather in the way that we look at an ROC curve in relation to the upper left-hand corner of the ROC plot (where *sensitivity* and *specificity* are both equal to one). The contour cuts the main diagonal of the plot at prior probability ${\hat{p}}_{c}$ = 0.5, when $\ln\left(1/{\hat{p}}_{c}\right)$ = ln(2) = 0.693 nats (Figure 8). This is the amount of information required to be certain of a binary outcome when the prior probability is equal to 0.5. However, the amount of information required to be certain of an outcome is not of any great practical significance in crop protection decision making. Rather than seeking certainty, a realistic objective is to develop predictors that provide enough information to enable better decisions, on average, than would be made with reliance only on prior probabilities. Thus we need to be able to consider predictor performance in terms of predictive values.

Perhaps the most important instrument available to the developer of a binary predictor is the placement of the threshold on the risk score scale [2,3,6,8]. This determines a predictor’s *sensitivity* and *specificity*, and consequently the likelihood ratios for + and − predictions. However, this does not guarantee predictor performance in terms of predictive values. ROC curve analysis and diagrammatic formats that characterize predictive values (or functions of them) are therefore complementary aspects of predictor evaluation and comparison. For example, the appropriate placement of the threshold on the risk score scale may be informed by knowledge of disease prevalence for the scenario in which the predictor is intended for application. This in turn affords an evaluation of likely performance—in terms of predictive values—for the predictor in operation. Sometimes, however, we may wish to compare predictors’ likely performances—perhaps in a novel scenario—when we are simply a potential user of the predictors in question, having had no development input but with access to the predictors’ ROC curve summary statistics. In both settings, the diagrammatic formats we have discussed have potential application. They lead to information graphs that are visually distinct but analytically linked. All give extra insight via the predictive values of disease forecasts. 

## Figures and Tables

**Table 1:** The prediction-realization table for a test with two categories of realized (actual) status (*c*, *nc*) and two categories of prediction (+, −). In the body of the table are the joint probabilities.

	Realization		
Prediction	*c*	*nc*	Row Sums
+	${\hat{p}}_{+\cap \;c}$	${\hat{p}}_{+\cap \;nc}$	${\hat{p}}_{+}$
−	${\hat{p}}_{-\cap \;c}$	${\hat{p}}_{-\cap \;nc}$	${\hat{p}}_{-}$
Column sums	${\hat{p}}_{c}$	${\hat{p}}_{nc}$	1

**Table 2:** Example data set. See [6,11] for full details.

Scenario	${\hat{p}}_{c}$	${\hat{p}}_{+}{|}_{\;c}$	${\hat{p}}_{-}{|}_{\;nc}$	${\hat{p}}_{c}{|}_{+}$	${\hat{p}}_{nc}{|}_{-}$
A	0.36	0.833	0.844	0.75	0.90
B	0.05	0.833	0.844	0.22	0.99
C	0.05	0.390	0.990	0.67	0.97
D	0.85	0.833	0.844	0.97	0.47
E	0.85	0.944	0.656	0.94	0.67

${\hat{p}}_{c}$: prior probability of an epidemic or for the need for a control intervention, estimated by disease prevalence. ${\hat{p}}_{+}{|}_{\;c}$: estimated probability of an actual epidemic being correctly predicted on using a test (as defined by a prediction-realization table). Referred to as *sensitivity*. ${\hat{p}}_{-}{|}_{\;nc}$: estimated probability of an actual non-epidemic being correctly predicted on using a test (as defined by a prediction-realization table). Referred to as *specificity*. ${\hat{p}}_{c}{|}_{+}$: estimated posterior probability of an epidemic given that one is predicted on using a test (as defined by a prediction-realization table). Referred to as *positive predictive value*. ${\hat{p}}_{nc}{|}_{-}$: estimated posterior probability of no epidemic given that one is not predicted on using a test (as defined by a prediction-realization table). Referred to as *negative predictive value*.
